# Prenatal, neonatal and postnatal factors and the developmental defects of dental enamel

**DOI:** 10.1590/1984-0462/2024/42/2022226

**Published:** 2023-08-25

**Authors:** Sandra Espíndola Lunardelli, Abelardo Nunes Lunardelli, Luiz Gustavo Teixeira Martins, Eliane Traebert, Jefferson Traebert

**Affiliations:** a Universidade do Sul de Santa Catarina, Palhoça, SC, Brasil.

**Keywords:** Enamel defects, Epidemiology, Children, Defeitos de esmalte, Epidemiologia, Crianças

## Abstract

**Objective:**

To estimate the prevalence of developmental defects in dental enamel and its possible association with prenatal, neonatal and postnatal conditions in six-year-old schoolchildren in a southern Brazilian municipality.

**Methods:**

A cross-sectional study was conducted involving 655 six-year-old schoolchildren. Sociodemographic and health data were collected through interviews with mothers and children’s oral examinations at schools. Multivariate analyses were performed using Poisson regression with robust estimator.

**Results:**

The prevalence of developmental defects of enamel was 44.0%. Demarcated opacities were the most prevalent, followed by diffuse opacities. Late pregnancy, maternal schooling less than eight years, female gender and child’s white skin color were independently associated with the prevalence of demarcated opacities.

**Conclusions:**

The prevalence of developmental defects in dental enamel was 44.0%. Late pregnancy, maternal schooling less than eight years, female gender and child’s white skin color were associated with the prevalences.

## RESUMO

**Objetivo:**

Estimar a prevalência de defeitos de desenvolvimento do esmalte dentário e sua possível associação com fatores pré-natais, neonatais e pós-natais em escolares de seis anos de idade em um município do sul do Brasil.

**Métodos:**

Foi conduzido um estudo transversal envolvendo 655 escolares de seis anos de idade. Os dados sociodemográficos e de saúde foram coletados por meio de entrevistas com as mães e exames bucais das crianças nas escolas. As análises multivariadas foram realizadas por meio de regressão de Poisson com estimador robusto.

**Resultados:**

A prevalência de defeitos de desenvolvimento do esmalte foi de 44,0%. As opacidades demarcadas foram as mais prevalentes, seguidas das difusas. Gravidez tardia, escolaridade materna inferior a oito anos, sexo feminino e cor da pele branca da criança foram independentemente associados à prevalência de opacidades demarcadas.

**Conclusões:**

A prevalência de defeitos de desenvolvimento do esmalte dentário foi de 44,0%. Gravidez tardia, escolaridade materna inferior a oito anos de estudo, sexo feminino e cor da pele branca da criança estiveram associados às prevalências.

## INTRODUCTION

Negative impacts occurring in the early stages of life may directly affect adult health due to permanent damage to cells and organs, and indirectly, interfering in their socioeconomic performance.^1^ During the development of dental structures, numerous etiologic agents can disturb the matrix formation and mineralization. These agents can affect a single tooth or can have a systemic nature, affecting groups of teeth. The ameloblasts, precursor cells of dental enamel, are highly specialized and extremely sensitive to local and systemic stimuli, and may produce defects in the enamel structure during its formation in the prenatal, neonatal and postnatal periods. The tooth enamel is the only hard tissue of the body that does not change. So, all changes in its structure that come from aggressions during its development will be permanently recorded.^2^

Developmental defects of enamel (DDE) are disturbances in hard tissues matrices and mineralization, originated during odontogenesis.^3^ When the ameloblast aggression occurs during the secretion phase of the enamel matrix or in the initial stage of the transition, a reduction in its thickness may occur, leading to hypoplasia. When the etiologic agent is acting during the maturation phase or in the final stage of the transition phase, it may result in a hypomineralization, also named as enamel opacity, due to its clinical aspect.^2^ Based on macroscopic appearance, these defects are classified as hypoplastic, diffuse opacities and demarcated opacities.^3^ Hypoplasia is a defect in which localized reduction in enamel thickness occurs. It can occur as fissures, grooves and partial or total absence of enamel over a considerable dentin surface. The affected enamel may be translucent or opaque. Diffuse opacity is a defect involving a change in the enamel translucency, which can be variable in different levels. The defective enamel has a normal thickness and when it erupts, it has a relatively smooth surface and a white coloration. It may have a linear, stained or confluent distribution, but there is no clear limit with the adjacent normal enamel. The demarcated opacity is a defect involving a change in the enamel translucency, variable in degrees. The defective enamel has a normal thickness with a smooth surface. It has a clear and distinct border with the adjacent normal enamel, and it can be white, cream, yellow or brown.^3^ DDE are important as strong predictors of dental caries.^4–6^ Populations affected by these alterations may require, as a priority, early preventive and curative interventions in relation to decays.

Thus, the aim of this study was to estimate the prevalence of DDE and to assess its association with prenatal, neonatal, and postnatal factors in six-year-old schoolchildren.

## METHOD

A cross-sectional study was carried out nested in a longitudinal study named *Coorte Brasil Sul* ^7^ involving six to seven-year-old schoolchildren born in 2009, and their families, living in the city of Palhoça in the southern Brazilian state of Santa Catarina. Children were enrolled in the first year of elementary school.

The sample size (n=664) was calculated with the following parameters: total population of 1,756 students, confidence level of 95%, unknown prevalence of DDE (p=50%), and a relative error of 3%. The selection of the sample was performed by simple random draw involving all six to seven-year-old schoolchildren from all 37 public and 19 private primary schools of Palhoça.

Children who presented the following three conditions at the same time were included in the study: born in 2009, enrolled in public or private schools in the municipality in 2015, and residents in the municipality of Palhoça.

Children with congenital, facial, or syndromic deformities (cleft lip, cleft palate, Down syndrome, hereditary ectodermal dysplasia, and cerebral palsy) and children whose family language was not Portuguese were excluded from the study. The lack of signature of the Free and Informed Consent Form by the parents, the non-assent by the child at the time of the oral examination, and the situation in which the mother was not found at home in three visits (one at the weekend) were considered as losses and refusals.

Data were collected through interviews, consultation of children’s health card (number of prenatal consultations, gestational age, delivery route, birth weight, congenital anomalies, and Apgar in the 1^st^ and 5^th^ minute), and clinical dental examinations. Interviews in households were carried out with the mother of the child. A pilot study was conducted in order to collect data in the households. Previously trained community health agents applied 120 questionnaires in the communities. No major changes were deemed necessary.

Children’s oral examinations were performed in schools by eight dentists and eight oral health auxiliaries. To diagnose and classify enamel changes, the Modified Developmental Defects of Enamel Index^3^ was used as follows:

1. “Demarcated Opacity” – defect involving alteration in enamel translucency. In this case, defective enamel is of normal thickness with a smooth surface. It has a clear, distinct boundary from adjacent normal enamel and may be white, cream, yellow or brown in color;
2. “Diffuse opacity” – defect involving alteration in enamel translucency, variable in levels. Defective enamel has normal thickness and when erupted has a relatively smooth surface and its color is white. It may have a linear, mottled, or confluent distribution, but there is no clear boundary with adjacent normal enamel;
3. “Enamel hypoplasia” – defect involving the enamel surface and associated with localized reduction in enamel thickness. It can occur in the form of pits (single or multiple, shallow or deep, diffuse or aligned, arranged horizontally on the surface of the tooth), in furrows (single or multiple, narrow or wide), or the absence (total or partial) of enamel over a considerable area of dentin. In this case, the affected enamel can be translucent or opaque.^3^

Children were examined in a classroom with natural light in addition to artificial environment lighting. A flat mouth mirror was used to observe DDE, and a periodontal probe was used only in cases of doubts about the presence of small fissures and grooves. They were examined without prophylaxis or previous dental brushing. The buccal, occlusal/incisal and lingual/palatal surfaces of all deciduous and permanent teeth were also examined.

The qualification and calibration of the examiners for the DDE were performed in 12 activity hours, using the *in-lux* method. All the examiners reached values of Kappa ≥0.65 in both intra-examiner and inter-examiner calibration.

The dependent variables were the prevalence and types of DDE in deciduous and permanent dentitions. The independent variables were:

1. Prenatal period: child gender, child’s skin color, relatives’ schooling when the child was born, teenage and late pregnancy, number of prenatal consultations, maternal smoking, alcohol and drugs use while pregnant, occurrence of infectious diseases, pneumonia, vaginal discharge, urinary infection, gestational diabetes, hypertension and heart diseases while pregnant;
2. Neonatal period: gestational age, delivery route, birth weight, congenital anomaly, and Apgar in the 1^st^ and 5^th^ minute;
3. Postnatal period: breastfeeding and time, child’s medical care intervention for more than two days, use of medication for more than 30 consecutive days, and use of antibiotics, occurrence of varicella, rubella, pneumonia, diarrhea, verminosis, tonsillitis, skin or ear infection, diabetes, heart diseases, acid reflux or anemia until two years of age.

Multivariate analyses were performed to identify independent relationships between studied variables. The model was composed of variables which p≤0.20 values were observed in the bivariate analysis. Poisson regression with robust estimator was used to estimate the prevalence ratios (PR) and their confidence intervals (CI) at the 95% accuracy level.

This study was approved by the Human Research Ethics Committee of the *Universidade do Sul de Santa Catarina* under the protocol number 38240114.0.0000.5369.

## RESULTS

Based on the information from the questionnaires and clinical examinations, 655 families were included in this study, from the initial sample (n=664), resulting in a response rate of 98.6%. Of the total, 50.2% of children were female and 82% were white.

At the time of the child’s birth, 49.6% of the mothers had no income, 31.5% had completed eight years of study, and 77.0% had white skin color. Regarding fathers, 4.6% had no income and 41.3% had completed primary school.

Teenage pregnancy occurred in 20.3% of cases, and 12.5% of the mothers had their children between 35 and 44 years old. Those who worked during pregnancy totalized 47.3%, and of them, 87.8% worked up to the seventh month of pregnancy. Prenatal consultations were performed by 98.1% of the pregnant women and 90.7% of them had six or more visits. Regarding the occurrence of infectious diseases during pregnancy, women reported one or more of the following pathologies: urinary tract infection (31.0%), vaginal discharge requiring treatment (23.7%), varicella (1.9%), toxoplasmosis (1.6%), pneumonia (1.2%), HIV/AIDS (0.5%), syphilis (0.5%), and cytomegalovirus, measles, rubella and tetanus (0.3% each of them). Hypertension and diabetes were found in 14.8% and 4.8% of women, respectively. The occurrence of heart disease was reported by 2.3% of pregnant women. Smoking, use of alcohol and drugs were found in 14.8%, 6.5% and 1.7%, respectively.

Cesarean sections occurred in 41.3% of deliveries. A total of 6.9% of study infants were premature, and 5.4% had low birth weight. A score lower than 8 at first-minute Apgar was verified in 7.8% of the children. Seven percent of infants had to be hospitalized in the first 10 days of life and 18.3% had neonatal jaundice. Respiratory problems up to the first 28 days of life were reported in 5.1% of infants and 3.3% required postpartum intubation.

Most children (91.9%) were breastfed, but 22.9% of them for less than six months. The use of medication for more than 30 consecutive days was observed in 16.5% of the children and 63.0% used antibiotics up to two years of age. Medical care intervention for more than two consecutive days occurred with 16.2% of the children. Regarding the occurrence of infectious diseases, children presented one or more of the following pathologies: diarrhea (59.3%), tonsillitis (54.7%), ear infection (39.1%), varicella (27.0%), pneumonia (22.5%), verminosis (22.5%), infection or skin wounds (17.8%) and rubella (1.1%). Reflux, anemia, heart disease and diabetes were found in 14.5%, 13.1%, 3.5% and 1.2% of children, respectively.

Duplicate clinical exams were performed in 40 schoolchildren (6.1% of the study population). Diagnostic reproducibility and concordance between examiners were high (Kappa >0.8).

The prevalence of DDE was 44.0% (95%CI 40.2–47.8). Demarcated opacities were the most common defects (31.1%), followed by diffuse opacities (19.1%) and enamel hypoplasia (6.9%). Combined defects were observed in 15.3% of cases. In deciduous dentition the prevalence was 21.7% and in the permanent dentition, 35.6%.

Only demarcated opacities showed statistically significant associations with independent variables (Tables 1 to 3). In the multivariate analysis, mothers who had their children between 35 and 44 years old had a 96% higher prevalence of demarcated opacities compared to younger mothers (PR=1.96; 95%CI 1.23–3.11; p=0.004). Likewise, children of mothers with lower levels of schooling presented a 53% higher prevalence (PR=1.53; 95%CI 1.05–2.22; p=0.026). Female children had a 44% higher prevalence of demarcated opacity (PR=1.44; 95%CI 1.01–2.05; p=0.042). White color skin children had 84% higher prevalence of demarcated opacity compared to non-white skin ones (PR=1.84; 95%CI 1.07–3.17; p=0.028) (Table 4).

**Table 1:** Association between the prevalence of demarcated opacity and prenatal variables.

		PR	95%CI	p-value
Child gender				
	Male	1.00		0.036
	Female	1.35	1.02–1.78	
Child skin color				
	Not white	1.00		0.060
	White	1.57	0.98–2.51	
Mother schooling when child was born (years)				
	≥8	1.00		0.050
	<8	1.35	1.00–1.82	
Father schooling when child was born (years)				
	≥8	1.00		0.700
	<8	1.06	0.77–1.46	
Late pregnancy				
	No	1.00		0.003
	Yes	1.72	1.20–2.46	
Teenage pregnancy				
	No	1.00		0.124
	Yes	0.76	0.53–1.00	
Number of prenatal consultations				
	6 or more	1.00		0.223
	Up to 5	0.71	0.41–1.23	
Use of alcohol while pregnant				
	No	1.00		0.710
	Yes	0.88	0.45–1.72	
Smoking while pregnant				
	No	1.00		0.269
	Yes	1.36	0.94–1.98	
Use of drugs while pregnant				
	No	1.00		0.325
	Yes	1.56	0.64–3.80	
Infectious diseases while pregnant				
	No	1.00		0.300
	Yes	1.46	0.72–2.96	
Pneumonia while pregnant				
	No	1.00		0.773
	Yes	0.82	0.20–3.28	
Vaginal discharge while pregnant				
	No	1.00		0.421
	Yes	0.87	0.61–1.23	
Urinary infection while pregnant				
	No	1.00		0.354
	Yes	1.16	0.85–1.57	
Diabetes while pregnant				
	No	1.00		0.614
	Yes	0.84	0.43–1.64	
Hypertension while pregnant				
	No	1.00		0.238
	Yes	1.25	0.86–1.82	
Heart diseases while pregnant				
	No	1.00		0.230
	Yes	0.50	0.16–1.55	

PR: prevalence ratio; CI: confidence interval.

**Table 2:** Association between the prevalence of demarcated opacity and neonatal variables.

		PR	95%CI	p-value
Gestational age (weeks)				
	37 to 42	1.00		0.895
	<37	0.89	0.49–1.88	
Route of delivery				
	Vaginal	1.00		0.272
	Cesarean	1.20	0.87–1.65	
Birth weight				
	≥2500 g	1.00		0.562
	<2500 g	0.81	0.40–1.65	
Congenital anomaly				
	No	1.00		0.222
	Yes	1.61	0.75–3.43	
1st minute Apgar				
	≥8	1.00		0.821
	<8	0.93	0.50–1.73	
5th minute Apgar				
	≥8	1.00		0.947
	<8	0.90	0.23–3.87	
Need for medical care intervention during the first 28 days				
	No	1.00		0.518
	Yes	0.81	0.42–1.54	
Respiratory problems during the first 28 days				
	No	1.00		0.288
	Yes	1.40	0.75–2.60	
Need for intubation				
	No	1.00		0.403
	Yes	0.74	0.36–1.50	
Neonatal jaundice				
	No	1.00		0.888
	Yes	1.02	0.72–1.45	

PR: prevalence ratio; CI: confidence interval.

**Table 3:** Association between the prevalence of demarcated opacity and postnatal variables.

		PR	95%CI	p-value
Breastfeeding				
	No	1.00		0.671
	Yes	0.90		
Time of breastfeeding (months)				
	≥6	1.00		0.086
	<6	1.33	0.96–1.84	
Need for medical care intervention for more than 2 days				
	No	1.00		0.769
	Yes	0.94	0.64–1.39	
Use of medication for more than 30 consecutive days				
	No	1.00		0.064
	Yes	0.69	0.47–1.02	
Use of antibiotics				
	No	1.00		0.701
	Yes	0.94	0.71–1.26	
Occurrence of varicella				
	No	1.00		0.352
	Yes	0.86	0.62–1.18	
Occurrence of rubella				
	No	1.00		0.933
	Yes	0.95	0.30–2.99	
Occurrence of pneumonia				
	No	1.00		0.373
	Yes	0.99	0.71–1.39	
Occurrence of diarrhea				
	No	1.00		0.403
	Yes	0.89	0.67–1.17	
Occurrence of verminosis				
	No	1.00		0.434
	Yes	1.14	0.82–1.59	
Occurrence of tonsillitis				
	No	1.00		0.280
	Yes	0.86	0.65–1.13	
Occurrence of skin infection				
	No	1.00		0.665
	Yes	0.92	0.64–1.32	
Occurrence of ear infection				
	No	1.00		0.404
	Yes	0.88	0.66–1.18	
Occurrence of diabetes				
	No	1.00		0.312
	Yes	1.07	0.34–3.34	
Occurrence of heart diseases				
	No	1.00		0.092
	Yes	0.43	0.16–1.15	
Occurrence of acid reflux				
	No	1.00		0.125
	Yes	0.72	0.47–1.09	
Occurrence of anemia				
	No	1.00		0.738
	Yes	1.07	0.71–1.63	

PR: prevalence ratio; CI: confidence interval.

**Table 4:** Results of multivariate analysis for demarcated opacity.

		PR_c_	95%CI	p-value	PR_a_	95%CI	p-value
Late pregnancy							
	No	1.00		0.003	1.00		0.004
	Yes	1.72	1.20–2.46		1.96	1.23–3.11	
Child’s gender							
	Male	1.00		0.036	1.00		0.042
	Female	1.35	1.02–1.78		1.44	1.01–2.05	
Mother’s schooling when the child was born							
	≥8 years	1.00		0.053	1.00		0.026
	<8 years	1.35	1.00–1.82		1.53	1.05–2.22	
Time of breastfeeding (months)							
	≥6	1.00		0.086	1.00		0.236
	<6	1.33	0.96–1.84		1.29	0.85–1.95	
Child’s skin color							
	Not white	1.00		0.060	1.00		0.028
	White	1.57	0.98–2.51		1.84	1.07–3.17	
Use of medication for more than 30 consecutive days							
	No	1.00		0.064	1.00		0.758
	Yes	0.69	0.47–1.02		1.08	0.68–1.71	
Occurrence of acid reflux							
	No	1.00		0.125	1.00		0.535
	Yes	0.72	0.47–1.09		0.85	0.51–1.42	

PR_c_: crude prevalence ratio; PR_a_: adjusted prevalence ratio.

## DISCUSSION

A general prevalence of 44% of DDE was found through this study. The literature^8,9^ showed similar rates in similar age groups. The highest prevalence (64.0%) was found in a Brazilian study^4^ with 8 to 12-year-old schoolchildren from public and private schools in Pelotas (RS). Another Brazilian study^10^, in Alfenas (MG), reported a DDE prevalence of 63.1% in children aged 8 to 11 years.

The present study showed a higher prevalence of demarcated opacities in relation to other defects. Despite the difficulty of comparing the studies more carefully, demarcated opacities were also more frequent in several works.^9–12^ Unlike other studies^4,13^, diffuse opacities were the most prevalent enamel defects, as shown in the Alfenas study^10^, where diffuse opacity was present in 36.7% of cases, demarcated opacity in 14.8%, and hypoplasia in 5.8%. A New Zealander study^8^ also showed a higher prevalence of diffuse opacities; however, the authors reported that all other studies conducted so far in New Zealand have identified demarcated opacities as the most prevalent type of enamel defect.

When studying specifically demarcated opacities — herein the most prevalent enamel defect —, it was found a statistically significant association with certain variables. Female children had higher prevalence when compared to boys. However, the literature is conflicting about gender. A study^14^ showed that girls were almost three times more likely to present DDE than boys, but no explanation was given about that. A study^15^ with Spanish children showed that boys had an increased risk of developing enamel defects. Another study^8^ also showed that boys had a higher risk and the authors suggested that it was due to the their higher nutritional requirements; they weigh more than girls, have greater muscle percentage and faster growth, so they tend to be more susceptible to enamel defects. On the other hand, most studies did not reveal statistically significant differences between genders.^8,16,17^

Child’s white skin color was associated with the greater occurrence of demarcated opacities. However, there is no explanatory hypothesis for this finding. In literature, it is very difficult to find studies about the relationship between ethnicity and enamel defects. A study^17^ from Singapore showed a higher occurrence of incisor-molar hypoplasia (IMH) in Malay than in Chinese children. This finding was hypothetically explained by the fact that Malay children are breastfed for a longer period of time. Prolonged breastfeeding plus the presence of toxic environmental products such as dioxin, present in breast milk, could be related to the occurrence of IMH.

Regarding socioeconomic profile, children whose mothers had less than eight years of schooling had a 53% higher prevalence of demarcated opacities. The level of education is a socioeconomic position marker widely used in numerous epidemiological surveys. This fact indicates it as an important predictor of morbimortality and health related behaviors. Low levels of schooling tend to lead to poorer working, income and housing, and less access to services and knowledge, with the potential to negatively influence health.^18^ Gretchen et al.^19^ pointed to the association between the pregnant women’s schooling years and the perinatal mortality rate, birth weight, and neurological abnormalities rates in children. They also reported that increased maternal education reduces significantly perinatal mortality and morbidity rates, and it is not due to reduced gestational problems, but because education is a good indicator of socioeconomic conditions.^20^ A study^21^ carried out in southern Brazil found an association between lower maternal schooling and monthly family income with the introduction of unhealthy and non-recommended foods in the child’s first year of life. This study reinforces the strong association between low socioeconomic status, morbidity and poorer health habits.

Some studies have shown association between low socioeconomic level and enamel defects. The Spanish study^15^ reported an association between DDE and a lower socioeconomic level. A Brazilian study^22^ found that low family income was observed in 85% of children with molar incisor hypomineralization from public schools and only 18% in children from private schools. Another study^16^ showed that DDE was more prevalent in children whose parents had lower level of schooling. Tourino et al.^22^ reported that the prevalence of DDE was associated with lower family income.

Pre, neo and postnatal conditions respond to the biological aspects that occurred in the first thousand days of a child’s life. Only mother’s age at conception was significantly associated with demarcated opacities. Children whose mothers aged between 35 and 44 years at the time of birth had a 96% higher prevalence of demarcated opacities. Literature is scarce in relation to studies that address the mother’s age at conception with DDE. A case-control study^16^ showed a higher prevalence of DDE in those children whose mothers were less than 24 years old at the time of the child’s birth. Thus, the possibility of comparison is impaired due to the lack of similar parameters of maternal age stratification and study design.

The risk of late pregnancy on woman’s and child’s health has been widely described in the obstetrical literature. Pregnancy in women over 35 years old are associated with a higher risk of complications, such as spontaneous abortion in the first trimester, chronic hypertension, pregnancy-specific hypertension, gestational diabetes, and previous placenta. A systematic review^23^ showed that children of mothers who had gestational diabetes presented an increased likelihood of general DDE, IMH and hypoplasia. There is no maternal age free of chromosomal disorders that affect the child. Nevertheless, it has been universally accepted that mother’s aged 35 years or older at birth implies an increased risk.^24^ Epidemiological data have demonstrated a direct relationship between maternal age and chromosomal abnormalities in children, including trisomy 21, which is strongly associated with defects in enamel development.^24^ In addition to this genetic factor, an explanatory hypothesis is that women over 35 years old may require a higher nutritional intake and parity, which is associated with a higher metabolic stress.^24^

Teenage pregnancy was not associated with the prevalence of enamel defects. The risks associated with adolescent pregnancy, such as prematurity, low birth weight, anemia, specific hypertensive disorder of pregnancy, and complications in delivery have been attributed to the biological immaturity of the adolescent. Although this study did not show a statistically significant association with birth weight, it is important to notice that a recent meta-analysis demonstrated that both preterm and low birth weight, especially very low birth weight, were associated with a higher risk of DDE in the primary dentition.^25^ Another meta-analysis^26^ detected an increased risk of developing DDE in preterm children with a higher risk in the primary dentition. Also, demarcated opacities and hypoplasia were associated with birth weight of 2,500g or less in a Brazilian study.^10^ It is currently believed that unfavorable environmental factors such as low education, emotional instability, undernutrition, nutritional deficiencies, anemia and smoking are determining factors of the main complications of teenage pregnancy.^27^ In addition, pregnant women under 18 present a lower risk of malformations and chromosomal alterations than women over 35 years old.^27^

Finally, the wide variation of the analyzed age groups and the great diversity of the methodological aspects adopted in the studies can be cited as complicating factors for the discussion of the present work. Factors can be, for example: the analyses of only deciduous teeth, only permanent or both together; DDE as a whole or only hypoplasia or opacities; the kind of lighting used in the clinical examination; previous tooth brushing and professional prophylaxis; and drying teeth performed before clinical examination. All these issues have the potential to modify the DDE prevalence. Another limitation specially related to the present work is the possible bias regarding the mother’s recall about prenatal, neonatal, and postnatal factors addressed in the study, which also has the potential to change the results. However, high diagnostic reproducibility and agreement between the examiners ensured reliability of the clinical data collected in this research.

The constant changes in the socioeconomic conditions of individuals and populations affect health conditions, reflecting different biological, economic, social, and psychological risks for the development of diseases.^28^ Public policies to promote and protect individual health, especially in the most critical periods of their lives, such as the transition to motherhood and early childhood, should be a priority. The lower level of the mother´s schooling when she gets pregnant and all the consequences of this situation contribute to the damages suffered by the child in the first thousand days of life, and is also related to the presence of DDE.

It can be concluded through this study that late pregnancy, less than eight years of maternal schooling, female gender and the child’s white skin color were associated with the prevalence of 44% of DDE.

## Data Availability

The database that originated the article is available with the corresponding author.
